# Alcohol Consumption During Pregnancy and Risk of Placental Abnormality: The Japan Environment and Children’s Study

**DOI:** 10.1038/s41598-019-46760-1

**Published:** 2019-07-16

**Authors:** Satoshi Ohira, Noriko Motoki, Takumi Shibazaki, Yuka Misawa, Yuji Inaba, Makoto Kanai, Hiroshi Kurita, Tanri Shiozawa, Yozo Nakazawa, Teruomi Tsukahara, Tetsuo Nomiyama, The Japan Environment & Children’s Study (JECS) Group, Toshihiro Kawamoto, Hirohisa Saito, Reiko Kishi, Nobuo Yaegashi, Koichi Hashimoto, Chisato Mori, Shuichi Ito, Zentaro Yamagata, Hidekuni Inadera, Michihiro Kamijima, Takeo Nakayama, Hiroyasu Iso, Masayuki Shima, Yasuaki Hirooka, Narufumi Suganuma, Koichi Kusuhara, Takahiko Katoh

**Affiliations:** 1 Center for Perinatal, Pediatric, and Environmental Epidemiology, Shinshu University School of Medicine, 3-1-1 Asahi, Matsumoto, Nagano, 390-8621 Japan; 2 Department of Obstetrics and Gynecology, Shinshu University School of Medicine, 3-1-1 Asahi, Matsumoto, Nagano, 390-8621 Japan; 3 Department of Pediatrics, Shinshu University School of Medicine, 3-1-1 Asahi, Matsumoto, Nagano, 390-8621 Japan; 4 Department of Preventive Medicine and Public Health, Shinshu University School of Medicine, 3-1-1 Asahi, Matsumoto, Nagano, 390-8621 Japan; 5 Department of Neurology, Nagano Children’s Hospital, 3100 Toyoshina, Azumino, Nagano, 399-8288 Japan; 6 University of Occupational and Environmental Health, 1-1 Iseigaoka, Yahatanishi-ku Kitakyushu, Fukuoka, 807-8555 Japan; 7 National Center for Child Health and Development, 2-10-1 Okura, Setagaya-ku, Tokyo 157-8535 Japan; 8 Hokkaido University, Kita 8, Nishi 5, Kita-ku, Sapporo, Hokkaido 060-0808 Japan; 9 Tohoku University, 2-1 Seiryo-machi Aoba-ku, Sendai, Miyagi 980-8575 Japan; 10 Fukushima Medical University, 1Hikariga-oka, Fukushima-shi, Fukushima, 960-1247 Japan; 11 Chiba University, 1-33 Yayoicho, Inage-ku, Chiba-shi, Chiba 263-8522 Japan; 12 Yokohama City University, 3-9 Fukuura, Kanazawa-ku, Yokohama, Kanagawa 236-0004 Japan; 13 University of Yamanashi, 1110 Shimokato, Chuo, Yamanashi 409-3898 Japan; 14 University of Toyama, 2630 Sugitani, Toyama-shi, Toyama 930-0194 Japan; 15 Nagoya City University, 1 Kawasumi, Mizuho-cho, Mizuho-ku, Nagoya, Aichi 467-8601 Japan; 16 Kyoto University, Yoshida-honmachi, Sakyo-ku, Kyoto 606-8501 Japan; 17 Osaka University, 2-2 Yamadaoka, Suita, Osaka 565-0871 Japan; 18 Tottori University, 86 Nishi-cho, Yonago, Tottori 683-8503 Japan; 19 Hyogo College of Medicine, 1-1 Mukogawa-cho, Nishinomiya, Hyogo 663-8501 Japan; 20 Kochi University, Okochokohasu, Nankoku, Kochi 783-8505 Japan; 21 Kumamoto University, 1-1-1 Honjo, Chuo-ku, Kumamoto 860-8556 Japan

**Keywords:** Epidemiology, Risk factors

## Abstract

There have been no large nationwide birth cohort studies examining for the effects of maternal alcohol use during pregnancy on placental abnormality. This study searched for associations between alcohol consumption and the placental abnormalities of placenta previa, placental abruption, and placenta accreta using the fixed dataset of a large national birth cohort study commencing in 2011 that included 80,020 mothers with a singleton pregnancy. The presence of placental abnormalities and potential confounding factors were recorded, and multiple logistic regression analysis was employed to search for correlations between maternal alcohol consumption during pregnancy and placental abnormalities. The overall rate of prenatal drinking until the second/third trimester was 2.7% (2,112). The prevalence of placenta previa, placental abruption, and placenta accreta was 0.58% (467), 0.43% (342), and 0.20% (160), respectively. After controlling for potential confounding factors, maternal alcohol use during pregnancy was significantly associated with the development of placenta accreta (OR 3.10, 95%CI 1.69-5.44). In conclusion, this large nationwide survey revealed an association between maternal drinking during pregnancy and placenta accreta, which may lead to excessive bleeding during delivery.

## Introduction

The placenta plays a crucial role in maternal-fetal exchange. Abnormalities of the placenta have been linked to such pregnancy complications as placental abruption, placenta previa, and placenta accreta^1^. Placental abruption is responsible for up to a third of all perinatal deaths^2^, mainly owing to the disruption of gestation length and fetal growth^3,4^. Placenta previa can also restrict fetal growth and cause preterm delivery and perinatal mortality^5^. Placenta accreta is associated with premature birth and excessive vaginal bleeding during delivery^6^.

By readily crossing the placenta, alcohol may play a role in vascular lesion development leading to unfavorable outcomes. Specifically, dose-dependent alcohol exposure produces placental vasoconstriction and lower placental weight^7,8^. Alcohol may also alter important embryonic processes to impair normal development and lead to morbidity or mortality^8,9^. In one survey, only 1.6% of pregnant women reported frequent drinking, but roughly 12.5% described having at least some alcohol in pregnancy^8^. Most studies largely corroborate associations between alcohol and adverse pregnancy outcomes, but there is a lack of consensus regarding the relationship between maternal alcohol use and placental abnormality. Salihu *et al*.^10^ and Aliyu *et al*.^11^ previously examined for associations between prenatal alcohol consumption and the occurrence of placenta-associated syndromes that included placenta previa and placental abruption, but neither study evaluated for placenta accreta. Even though these previous studies had sufficient sample size, alcohol exposure might be underreported, biasing study results toward the null.

This large birth cohort study^12^ was conducted with two main goals: (1) clarify the effect of prenatal alcohol consumption on the risk of placenta previa, placental abruption, and placenta accreta, and (2) assess for dose dependencies between alcohol use and placental abnormality.

## Results

A total of 80,020 mothers with singleton births who completed the questionnaire were available for analysis. The overall rate of prenatal drinking until the second/third trimester was 2.7% (2,112). The prevalence of placenta previa, placental abruption, and placenta accreta were 0.58% (467), 0.43% (342), and 0.20% (160), respectively.

Table 1 summarizes the participants’ characteristics and alcohol exposure stratified by placental abnormalities (no placental abnormalities [controls], placenta previa, placental abruption, and placenta accreta). There were significant differences among the groups with regard to maternal age, past history of cesarean section, recurrent pregnancy loss, means of pregnancy, and smoking status. Significant differences also existed for maternal drinking status.

**Table 1:** Characteristics of participants with or without placental abnormality.

Variable	No placental abnormality (controls)	Placenta previa	Placental abruption	Placenta accreta	*P*
Participants, n	79,051	467	342	160	
Maternal age at delivery, years (mean ± SD)	31.2 ± 5.1	33.5 ± 4.7 ^b^	32.0 ± 5.2 ^b c^	32.3 ± 5.2^b^	<0.001^a^
Maternal age group, n (%)					
<20 years	674 (0.9)	0 (0.0)	5 (1.5)	0 (0.0)	
20–34 years	56,798 (71.8)	250 (53.5)	216 (63.2)	96 (60.0)	
35 + years	21,579 (27.3)	217 (46.5)	121 (35.4)	64 (40.0)	<0.001
Maternal BMI before pregnancy, kg/m^2^ (mean ± SD)	21.2 ± 3.3	21.3 ± 3.2	21.6 ± 3.7	21.6 ± 3.4	0.10^a^
Maternal BMI group					
Underweight (BMI < 18.5), n (%)	12,781 (16.2)	66 (14.1)	57 (16.7)	25 (15.6)	
Normal weight BMI 18.5–24.9), n (%)	57,911 (73.3)	350 (74.9)	239 (69.9)	115 (71.9)	
Overweight (BMI 25.0+), n (%)	8,359 (10.6)	51 (10.9)	46 (13.5)	20 (12.5)	0.51
Parity					
Multiparous, n (%)	45,402 (57.4)	276 (59.1)	193 (56.4)	96 (60.0)	0.78
Past history of cesarean section, n (%)	6,968 (8.8)	56 (12.0)	47 (13.7)	8 (5.0)	<0.001
Recurrent pregnancy loss, n (%)	813 (1.0)	11 (2.4)	3 (0.9)	2 (1.3)	0.043
Means of pregnancy for current birth, n (%)					
Spontaneous	73,967 (93.6)	394 (84.4)	310 (90.6)	123 (76.9)	
Ovulation induction through medication	2,112 (2.7)	17 (3.6)	7 (2.0)	3 (1.9)	
Artificial insemination or *in-vitro* fertilization	2,972 (3.8)	56 (12.0)	25 (7.3)	34 (21.3)	<0.001
Smoking during pregnancy, n (%)	3,572 (4.5)	17 (3.6)	26 (7.6)	14 (8.8)	0.002
Drinking during pregnancy, n (%)	2,012 (2.5)	10 (2.1)	8 (2.3)	13 (8.1)	<0.001
Maternal drinking frequency, n (%)					
Infrequent (<3 days per week)	1438 (1.8)	6 (1.3)	4 (1.2)	9 (8.3)	
Frequent (3+ days per week)	574 (0.3)	4 (0.9)	4 (1.2)	4 (2.5)	0.001
Maternal drinking amount, n (%)					
Low (<1.5 drinks per week)	1508 (1.9)	6 (1.3)	4 (1.2)	10 (6.3)	
High (1.5+ drinks per week)	504 (0.6)	4 (0.9)	4 (1.2)	3 (1.9)	0.001

^a^Differences in maternal age and BMI were assessed with one-way repeated measures of ANOVA followed by post-hoc (Bonferroni) testing.

^b^*P-*value < 0.001 vs. group of no placental abnormality.

^c^*P-*value < 0.001 vs. group of placental previa.

In multivariate logistic regression analysis after adjustment for covariates, we observed no remarkable differences in alcohol consumption during pregnancy as compared with controls for placenta previa or placental abruption. However, maternal drinking was significantly associated with placenta accreta (adjusted odds ratio [aOR] 3.03, 95% confidence interval [CI] 1.69–5.44). Regarding the frequency of alcohol consumption, infrequent (<3 days a week) and frequent (3+ days a week) drinking were also related to the incidence of placenta accreta (aOR 3.02, 95%CI 1.52–5.99 and aOR 3.06, 95%CI 1.11–8.48, respectively). Focusing on the amount of alcohol consumption, we observed significant differences for a low amount (<1.5 drinks a week) of drinking as compared with controls for placenta accreta (aOR 3.18, 95%CI 1.65–6.11). There were no associations between high drinking amounts during pregnancy and placental abnormalities (Table 2), although increases in the frequency (*P* < 0.001) and dose (*P* = 0.001) of drinking indicated a tendency for more frequent placenta accreta onset (Table 2).

**Table 2:** Multivariate logistic regression analysis for placental abnormalities versus controls.

Variable	Placenta previa (n = 467)				Placental abruption (n = 342)				Placenta accreta (n = 160)			
	cOR (95%CI)	*P*	aOR (95%CI)	*P*	cOR (95%CI)	*P*	aOR (95%CI)	*P*	cOR (95%CI)	*P*	aOR (95%CI)	*P*
**Drinking alcohol during pregnancy**	0.84 (0.45–1.57)	0.58	0.85 (0.45–1.61)	0.63	0.81 (0.45–1.85)	0.81	0.85 (0.42–1.73)	0.65	3.39 (1.92–5.98)	<0.001	3.03 (1.69–5.44)	<0.001
**Maternal drinking frequency status**												
Non-drinkers (reference)	1.00		1.00		1.00		1.00		1.00		1.00	
Infrequent (<3 days per week)	0.70 (0.31–1.58)	0.39	0.72 (0.32–1.61)	0.42	0.64 (0.24–1.72)	0.38	0.63 (0.23–1.69)	0.36	3.28 (1.67–6.44)	0.001	3.02 (1.52–5.99)	0.002
Frequent (3+ days per week)	1.18 (0.44–3.15)	0.75	1.20 (0.44–3.25)	0.72	1.61 (0.59–4.32)	0.35	1.33 (0.49–3.62)	0.58	3.65 (1.35–9.89)	0.011	3.06 (1.11–8.48)	0.031
*P* for trend		0.78		0.84		0.84		0.94		<0.001		<0.001
**Maternal drinking amount status**												
Non-drinkers (reference)	1.00		1.00		1.00		1.00		1.00		1.00	
Low (<1.5 drinks per week)	0.67 (0.30–1.50)	0.33	0.68 (0.30–1.53)	0.35	0.61 (0.23–1.64)	0.33	0.59 (0.22–1.60)	0.30	3.48 (1.83–6.61)	<0.001	3.18 (1.65–6.11)	0.001
High (1.5+ drinks per week)	1.34 (0.50–3.59)	0.56	1.39 (0.51–3.76)	0.52	1.83 (0.68–4.93)	0.23	1.52 (0.56–4.14)	0.42	3.12 (0.99–9.82)	0.052	2.61 (0.81–8.40)	0.11
*P* for trend		0.85		0.91		0.77		0.99		<0.001		0.001

Multiple logistic regression model was adjusted for maternal age, BMI before pregnancy, menstrual abnormality, recurrent pregnancy loss, parity, history of delivery (including cesarean section and artificial abortion), means of pregnancy, smoking habit, complications during pregnancy (including antiphospholipid antibody syndrome, maternal infection, diabetes mellitus/gestational diabetes mellitus, and hypertensive disorder of pregnancy), and medications during pregnancy (including antibiotics, iron supplements, folic acid supplements, and therapy for recurrent pregnancy loss).

## Discussion

This study represents the first Japanese nationwide birth cohort study assessing the influence of maternal alcohol consumption in pregnancy on the placental abnormalities of placenta previa, placental abruption, and placenta accreta. This large study indicates a significant association between maternal drinking during pregnancy and the incidence of placenta accreta.

In this nationwide self-reported survey, 2.7% of mothers responded to have consumed alcohol even after awareness of their pregnancy. Previous Japanese birth cohort studies described higher rates of drinking (11.8%^13^ and 13.4%^14^), which might have been due to our definition of drinkers who answered they drank up to the second/third trimester and exclusion of past drinkers who quit drinking early in pregnancy. Although this study included very few frequent or heavy drinkers among responders, dose-response and frequency-response trends were found between alcohol consumption and the development of placenta accreta.

Placenta previa, placental abruption, and placenta accreta were found to complicate 0.58%, 0.43%, and 0.20%, respectively, of singleton births, with prevalence rates similar to those of earlier reports. However, rates can differ among regions and races^15–23^.

Verification of sufficient analytical power was confirmed for the JECS cohort. In this scenario, testing a hypothesis for a disorder with a 0.1% prevalence, such as Down syndrome, 2.0 relative risk, and 0.05 alpha error using a sample in which the rate of individuals with high exposure to the studied chemical substance is 25% requires a cohort of 64,536 participants for a statistical power of 80%^12^. The JECS sample size exceeded this number.

Previous studies showed that maternal alcohol consumption increased the risk of placental abruption, but not of placenta previa^10,11^. Salihu *et al*.^10^ detected a significant positive correlation between alcohol consumption during pregnancy and placental abruption, although placenta previa was not remarkably linked to alcohol use during pregnancy. They also uncovered a J-shaped increase curve for placental abruption with increasing prenatal alcohol use. Similarly, Aliyu *et al*.^11^ found that mothers who consumed alcohol during pregnancy had an elevated risk of placental abruption, but not placenta previa. Yang *et al*.^16^ demonstrated that the effects of maternal drinking were stronger on placental abruption than on placenta previa. Unlike the present report, however, placenta accreta was not included in the above studies. Our findings revealed a significant association between maternal alcohol consumption during pregnancy and the outcome of placenta accreta, with none for placenta previa or placental abruption. In this regard, it provides new information on the relationship between placental abnormalities and drinking.

Maternal alcohol exposure during pregnancy may be a significant risk factor for placenta accreta, but the pathway of this phenomenon is not fully understood. Trophoblast invasion into the uterus is a key process during human placentation. In mice, perigestational alcohol exposure at organogenesis induced oxidative stress in the myometrium and trophoblast-decidual tissue, mainly affecting cells and macromolecules of trophoblasts and decidual tissues associated with placental formation^24^. Han *et al*.^25^ revealed that exposure to ethanol augmented the expression of nonmuscle myosin heavy chain-II (NMHC-II) associated with cell migration in human first-trimester trophoblast cell line HTR-8/SVneo extravillous trophoblast cells. In mouse models, they also demonstrated that acute ethanol exposure induced significant upregulation of NMHC-IIB expression in all regions of the placenta compared with control placental tissue. These findings might partially explain the mechanism underlying a predisposition to placenta accreta by maternal alcohol use.

In this study, the incidence of prior cesarean section in the accreta group was 5% and less than that in normal controls. We did not observe a significant positive relationship between placenta accreta and uterine scarring from previous cesarean delivery. Prior cesarean delivery was reported as a risk factor for placenta accreta or accreta with previa^20,26,27^. Other developed countries have a higher rate of cesarean delivery (20–29%)^28–30^ than in Japan, which tends to increase with the incidence of placenta accreta^26^. In the present investigation, the incidence of previous cesarean delivery was 12.3% for placenta accreta complicated with other placental abnormalities. The exclusion of cases with multiple placental abnormalities and the low rate of cesarean section in this cohort study were considered the reasons for a lower incidence of cesarean section in placenta accreta.

This study has several limitations. The data regarding alcohol consumption were collected from self-reported questionnaires and therefore subjective. Since the participants were part of a long-term cohort study, some selection bias might have been included. This study also did not account for regional differences in drinking habits. Another limitation was the lack of detailed information on the diagnosis of placental abnormalities because obstetricians recorded only the presence of abnormalities at birth from medical records; there may have been differences in the diagnostic criteria and degree of placental abnormality. Thus, misclassifications causing a bias towards the null value could have occurred. In the protocol of the JECS, misclassifications were regarded as equal to environmental toxin exposure since the raters were blinded to assessments^12^. Such misclassifications might have expanded the 95%CI.

In spite of the above limitations, this first study using a dataset from a Japanese nationwide birth cohort survey evaluated the impact of alcohol consumption in pregnancy while controlling for confounders identified by previous reports. It provides crucial evidence on the adverse effects of maternal alcohol exposure on placental abnormality.

In conclusion, this investigation uncovered a significant association between maternal alcohol consumption during pregnancy and placenta accreta, but not placenta previa or placental abruption. Although the study contained few frequent or heavy drinkers among responders, it detected dose-response as well as frequency-response tendencies of alcohol consumption on the development of placenta accreta. Further investigations are warranted to clarify the mechanisms underlying the detrimental effects of alcohol on placental pathogenesis.

## Materials and Methods

### Study design and participants

The data used in this investigation were adopted from the Japan Environment and Children’s Study (JECS), an ongoing cohort study commencing in January 2011 to assess the impact of environmental factors on children’s health.

**Figure 1:**
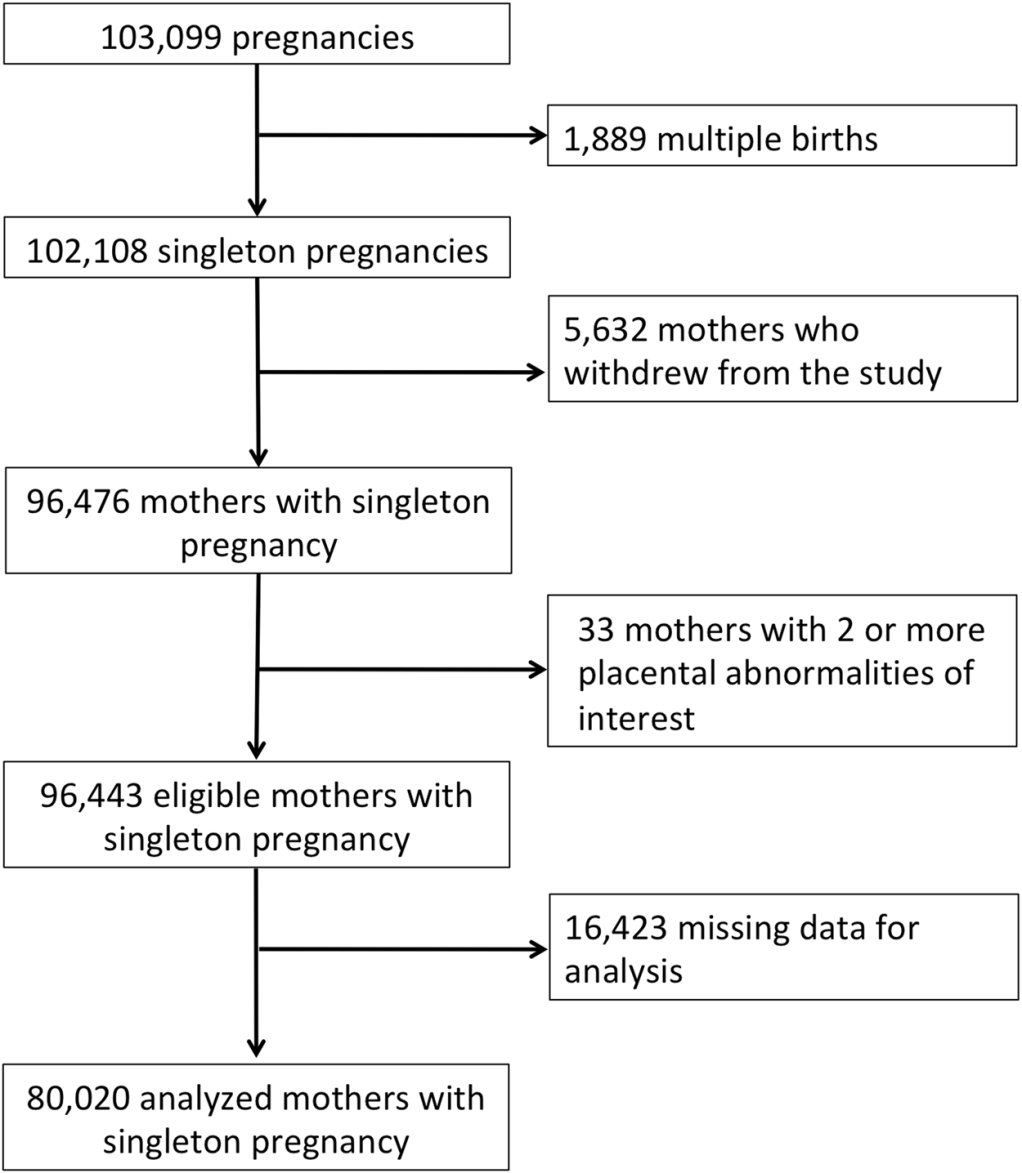
Case selection flowchart.

In the JECS, pregnant women were enrolled between January 2011 and March 2014 under the following inclusion criteria: (1) living in the study region at the time of enrollment, (2) delivery expected later than August 1, 2011, and (3) able to understand Japanese and fill out the self-administered survey. Details on the JECS project are available elsewhere^31,32^. The current study employed the “jecs-ag-20160424” dataset that was issued in June 2016 containing information on 96,476 mothers who had a singleton pregnancy. We analyzed data regarding alcohol consumption habits self-described by respondents during the second/third trimester, worded as follows: (1) no alcohol consumption, (2) quit drinking before pregnancy, (3) quit drinking during early pregnancy, and (4) drank during pregnancy. Subjects responding as (4) were further asked to describe their frequency and type of alcohol consumption. Information from medical record transcriptions regarding additional pregnancy details and medical history were used as other covariates. Of all the mothers who began the study, 80,020 (77.6%) completed the questionnaire (Fig. 1).

The Ministry of the Environment’s Institutional Review Board on Epidemiological Studies, and the Ethics Committees of all participating institutions approved the JECS protpcol^31^. The JECS was conducted in accordance with the Helsinki Declaration and other nationally valid regulations and guidelines. Written informed content was obtained from each participant.

### Data collection

Information on the alcohol consumption habits of mothers was collected during the second/third trimester of pregnancy from self-reported questionnaires. Maternal anthropometric data before pregnancy, complications and medication during pregnancy that included placental abnormalities, hypertensive disorders of pregnancy (HDP), and diabetes mellitus/gestational diabetes mellitus (DM/GDM), and a history of previous pregnancy were collected by the subjects’ obstetricians. Pre-pregnancy body mass index (BMI) was used to evaluate maternal weight status and was calculated according to World Health Organization Standards (body weight [kg]/height [m]^2^).

### Outcomes, exposure, and covariates

The main outcomes of interest were placenta previa, placental abruption, and placenta accreta. Placenta previa was judged as the placenta being attached to the uterine wall covering the internal os. This obstetric complication typically occurs in the late first trimester and may resolve later in pregnancy from so-called placental migration^33^. Placental abruption was determined as all or a part of the placenta pulling away from the uterine wall to hinder fetal blood and oxygen delivery. Small abruptions are able to resolve spontaneously, although larger ones may result in fetal distress or death^11^. We defined placenta accreta as abnormal uterine musculature attachment or invasion of placental tissue. Severe postpartum bleeding may result from failure of placental separation from the uterus after delivery, which has been associated with higher maternal morbidity and mortality^6^. We excluded 33 participants who became complicated with 2 or more placental abnormalities.

Alcohol consumption was assessed with a semi-quantitative food frequency questionnaire that contained a list of foods with standard portion sizes commonly consumed in Japan^34^. Regarding alcohol consumption, respondents indicating that they continued drinking throughout pregnancy were asked how often they drank and how many of what drinks they consumed. The frequency of maternal drinking during pregnancy was assessed by the questionnaire item, “Please choose an item that best describes your current drinking frequency”, and was grouped as follows: “hardly drank”, “once to three times a month”, “once to twice a week”, “three to four times a week”, five to six times a week”, and “drank everyday”. Alcohol content values for each beverage (Japanese sake, Japanese distilled spirits, beer, whiskey, and wine) were summated to calculate the total exposure amount of ethanol (g/week). We calculated the amount of ethanol as follows; 180 ml of Japanese sake contained 23 g of ethanol, 180 ml of distilled spirits contained 36 g of ethanol, a large bottle of beer (633 ml) contained 23 g of ethanol, 30 ml of whiskey contained 10 g of ethanol, and 60 ml of wine contained 9 g of ethanol. Based on previously published reports^11^, we divided drinkers into infrequent drinkers (<3 days per week) and frequent drinkers (3 + days per week). We also categorized drinkers into the following absolute alcohol amount categories: low (<1.5 drinks/week) and high (1.5 + drinks/week)^35^. The alcohol amount category of “low” included drinkers who chose the item of “hardly drank”. One standard drink was estimated to contain 14 g of ethanol^35,36^. We used non-drinking mothers as controls.

Demographic covariates included maternal age, smoking habit, and pre-pregnancy BMI. Obstetric and medical variables, such as parity, past history of cesarean section and artificial abortion, HDP, DM/GDM, and other complications and medications during pregnancy were also evaluated.

### Statistical analysis

All statistical analyses were performed using SPSS statistical software version 24 (SPSS Inc., Chicago, Illinois). Differences in maternal age and pre-pregnancy BMI among the types of placental abnormalities were assessed by one-way repeated measures of analysis of variance (ANOVA) followed by post-hoc (Bonferroni) testing. We categorized all continuous and ordinal variables, such as maternal age (<20, 20–34, and 35+ years), pre-pregnancy BMI (<18.5, 18.5–24.9, and 25+ kg/m^2^), and past parturient history (number of deliveries, cesarean section, and artificial abortion). Analysis of variance and chi-square tests were conducted to compare covariates between groups stratified by category as well as the frequency or amount of alcohol consumption. We used logistic regression models to generate crude odds ratios (cORs) and aORs and their 95%CIs. The selection of covariates in our models was done *a priori* according to published literature and biologic likelihood. We estimated the effect of alcohol consumption to adjust for maternal background, including age (<35 and 35+ years), pre-pregnancy BMI, smoking habit, maternal obstetric information (menstrual abnormality, recurrent pregnancy loss, parity, history of artificial abortion or cesarean section, means of this pregnancy), pregnancy complications such as anti-phospholipid antibody syndrome, maternal infection, HDP (mild and severe), and DM/GDM, and medications including antibiotics, iron supplements, folic acid supplements, and treatment for recurrent pregnancy loss. A *P*-value of <0.05 was considered statistically significant.
